# Comparing three forms of early intervention for youth with borderline personality disorder (the MOBY study): study protocol for a randomised controlled trial

**DOI:** 10.1186/s13063-015-1001-x

**Published:** 2015-10-21

**Authors:** Andrew Chanen, Henry Jackson, Sue M. Cotton, John Gleeson, Christopher G. Davey, Jennifer Betts, Sophie Reid, Katherine Thompson, Louise McCutcheon

**Affiliations:** Orygen, the National Centre of Excellence in Youth Mental Health, Melbourne, Australia; Centre for Youth Mental Health, The University of Melbourne, Melbourne, Australia; Orygen Youth Health, Northwestern Mental Health, Melbourne, Australia; Melbourne School of Psychological Sciences, The University of Melbourne, Melbourne, Australia; School of Psychology, Faculty of Health Sciences, Australian Catholic University, Melbourne Campus, Melbourne, Australia; Murdoch Children’s Research Institute, Melbourne, Australia

**Keywords:** Borderline personality disorder, Youth, Early intervention, Psychotherapy, Controlled trial, Cognitive analytic therapy, Befriending, Psychiatry

## Abstract

**Background:**

Borderline personality disorder is a severe mental disorder that usually has its onset in youth, but its diagnosis and treatment are often delayed. Psychosocial ‘early intervention’ is effective in improving symptoms and behaviours, but no trial has studied adaptive functioning as a primary outcome, even though this remains the major persistent impairment in this patient group. Also, the degree of complexity of treatment and requirements for implementation in mainstream health services are unclear.

The primary aim of this trial is to evaluate the effectiveness of three forms of early intervention for borderline personality disorder in terms of adaptive functioning. Each treatment is defined by combining either a specialised or a general service delivery model with either an individual psychotherapy or a control psychotherapy condition.

**Methods/design:**

The study is a parallel-group, single-blind, randomised controlled trial, which has randomised permuted blocking, stratified by depression score, sex and age. The treatments are: (1) the specialised Helping Young People Early service model plus up to 16 sessions of individual cognitive analytic therapy; (2) the Helping Young People Early service plus up to 16 sessions of a control psychotherapy condition known as ‘befriending’; (3) a general youth mental health care model plus up to 16 sessions of befriending. Participants will comprise 135 help-seeking youth aged 15–25 years with borderline personality disorder. After baseline assessment, staff blind to the study design and treatment group allocation will conduct assessments at 3, 6, 12 and 18 months. At the 12-month primary endpoint, the primary outcome is adaptive functioning (measures of social adjustment and interpersonal problems); secondary outcomes include measures of client satisfaction, borderline personality disorder features, depression and substance use.

**Discussion:**

The results of this trial will help to clarify the comparative effectiveness of a specialised early intervention service model over and above general youth mental health care, along with the contribution of individual cognitive analytic therapy over and above specialised general clinical care in early intervention for borderline personality disorder. Consequently, the findings will also inform the level of training and competency required for effective delivery of early intervention services.

**Trial registration:**

Registered with the Australian New Zealand Clinical Trial Registry ACTRN12610000100099 on 1 February 2010.

## Background

Borderline personality disorder is a severe mental disorder that is characterised by a pervasive pattern of impulsivity and instability in emotion regulation, interpersonal relationships and self-image [1]. Borderline personality disorder affects up to 3 % of the population [2, 3] and is common in psychiatric practice, affecting around one in five outpatients [4]. It is associated with high levels of health resource usage [5] and with adverse long-term outcomes that include severe and persistent functional disability [6], high family and carer burden [7], physical ill health [8, 9] and premature mortality [10], including a suicide rate of 8 % [11].

Although borderline personality disorder usually has its onset in the period between puberty and emerging adulthood (young people), diagnosis is often delayed and specific treatment for the disorder is rarely offered at this early stage [12, 13]. When treatment is eventually offered, functional impairment and iatrogenic complications are already entrenched, limiting the effectiveness of treatment, especially upon functional outcomes [6, 14].

The past two decades has seen evidence establishing that personality disorder diagnoses can be made prior to age 18 years [15] and that there is no point of rarity with personality disorder in adults [12]. Moreover, numerous reviews (for example, [16–19]) have concluded that borderline personality disorder is a unitary construct across the life course in terms of phenomenology, structure, stability, validity and morbidity. This knowledge has sanctioned the diagnosis of personality disorder in young people [20, 21] and has led to the first wave of randomised controlled trials (RCTs) of psychosocial treatments for borderline personality disorder in adolescents, which are summarised elsewhere [12, 22]. These treatments include cognitive analytic therapy (CAT) [23] delivered within the Helping Young People Early (HYPE) program [24], Emotion Regulation Training (ERT) [25, 26], Mentalisation-Based Treatment for Adolescents (MBT-A) [27], and Dialectical Behaviour Therapy for Adolescents (DBT-A) [28].

Overall, these trials have emphasised the importance of diagnosing and treating borderline personality disorder in young people, and the key findings support the effectiveness of psychosocial treatments for adolescents with either the features of, or full-syndrome, borderline personality disorder. All treatments, including good clinical care (GCC), treatment as usual (TAU) and enhanced usual care (EUC), were associated with improvement on outcome measures that included internalising and externalising psychopathology, depressive symptoms, borderline personality disorder features, quality of life, deliberate self-harm, and suicidal ideation. However, the structured interventions (CAT, GCC, MBT-A, DBT-A) generally outperformed TAU or EUC, except in the case of ERT.

These findings largely mirror those in adults with borderline personality disorder, where various specialist treatments seem to have similar effects despite distinct theories and interventions [14]. It remains unclear what role specific components of treatment (service model versus individual psychotherapy) might play in outcome in these trials. Such findings raise the challenging idea that specialised individual psychotherapy might not be the *sine qua non* of treatment for borderline personality disorder [12]. These psychotherapies are especially resource-intensive in terms of training, supervision and implementation, and this has important implications for the cost effectiveness of treatments. It is unclear what level of treatment complexity is required for effective intervention and, therefore, how treatments might be implemented in mainstream health services to address the high prevalence of borderline personality disorder. Therefore, it is important to control for some of the ‘common factors’ of psychotherapy, such as time in therapy, participant expectations and positive experiences of therapy.

These trials have used a range of comparison treatments, including manualised GCC [29], non-manualised TAU [25–27], and non-manualised EUC [28]. The absence of manuals or checks for treatment adherence or competency for TAU and EUC represent significant limitations to these studies. There was likely to be wide variation in practices among these treatments, and it cannot be assumed that TAU or EUC are equivalent or have similar effects. Moreover, TAU might include poor practice [29] or act as a *nocebo*, whereby potentially harmful effects arise from a placebo [22], which might inflate the effect size for the active treatment. Also, of the treatments studied, CAT, GCC, MBT-A and DBT-A are systematised interventions with individual and family components that were delivered within a comprehensive mental health service. However, this service model was manualised only for CAT and GCC.

A key point of difference among the studies is that the HYPE/CAT and ERT studies were primarily focused upon early intervention for borderline personality disorder, whereas the MBT-A and DBT-A studies were primarily focused upon suicidal and self-harming behaviour. It is unclear whether the patient samples in these studies are comparable. Not all treatment with young people is “early intervention” [12], and it is possible that participants were enrolled at different phases of illness (first-presentation patients through to those with enduring disorder). Also, the above RCTs combined ‘subthreshold’ individuals with features of borderline personality disorder who did not meet the full DSM-5 diagnosis (<5 diagnostic criteria) and those with the full-syndrome disorder (5–9 diagnostic criteria). This combines ‘indicated’ prevention with early intervention [13], and it is unclear whether treatment effects might differ among the two forms of intervention. Moreover, these RCTs restricted their inclusion criteria to adolescents (aged 12 to 19 years). Given the continuity of borderline personality disorder across adolescence and emerging adulthood [13], early intervention trials need to include new onset cases across the typical age of onset for the disorder.

Another key limitation is that the primary outcome measures for all these RCTs generally focus on symptoms and behaviours. It has become evident among young people and adults that these features of borderline personality disorder naturally attenuate over time but that impairments in adaptive functioning (vocational functioning and interpersonal relationships) remain stably poor, have proven to be refractory to treatment and should be a primary focus of treatment [12, 14].

### Aims and hypotheses

The overall purpose of this study is to evaluate the effectiveness of three forms of early intervention for adolescents and emerging adults (youth) with borderline personality disorder. The three early interventions vary in their degree of complexity, as determined by different components of service delivery (HYPE versus a general youth mental health service (YMHS)) and individual psychotherapy (CAT versus befriending (BEF)). The three early interventions are (in order of increasing complexity): YMHS + BEF, HYPE + BEF, and HYPE + CAT. The findings of this trial will help to clarify the level and complexity of services and treatments required for effective early intervention for borderline personality disorder.

The specific study aims are to: (i) elucidate whether a specialist early intervention service (HYPE) in addition to CAT (HYPE + CAT) will lead to superior outcomes as compared with the HYPE + BEF and the YMHS + BEF interventions; and (ii) determine whether there is a difference in effectiveness between the HYPE and YMHS service models.

The primary hypothesis is that at the 12-month primary endpoint participants receiving HYPE + CAT will have better outcomes on the primary (social adjustment and interpersonal problems) and secondary (borderline personality disorder symptoms, suicidal ideation, self-injury, depression, alcohol and substance use) outcome measures than those in the HYPE + BEF and YMHS + BEF interventions.

A secondary hypothesis is that at the 12-month primary endpoint, those treated with the HYPE model will have better outcomes on primary and secondary outcome measures than those young people being treated with the YMHS model.

## Methods

### Study design

This study is a parallel group, single-blind RCT comparing three groups: HYPE + CAT, HYPE + BEF, and YMHS + BEF. A full factorial design ( that is, including a fourth treatment arm comprising YMHS + CAT) was not implemented in order to meaningfully differentiate HYPE from YMHS. The HYPE service model incorporates the theory and principles of CAT, without CAT necessarily being used as an individual therapy. This is a key point of differentiation from usual youth mental health service models. If CAT were introduced at the YMHS site it would change usual YMHS practice (that is, the YMHS would represent a specialised rather than generalised service), thus limiting the external validity of this intervention.

**Fig. 1:**
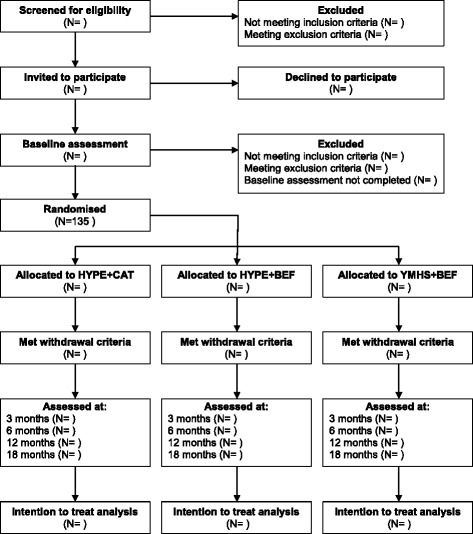
CONSORT flow diagram for Monitoring Outcomes of Borderline personality disorder in Youth (MOBY) trial. Flow diagram of MOBY study design

The study design was developed in accordance with Good Clinical Practice (GCP) Guidelines and Standard Protocol Items; Recommendations for Interventional Trials (SPIRIT [30]). Figure 1 summarises the trial design. The trial is registered with the Australian New Zealand Clinical Trial Registry (ACTRN12610000100099) and has been approved by the Melbourne Health Human Research Ethics Committee (HREC2010.055).

### Setting

The HYPE + CAT and HYPE + BEF treatments are being delivered at the HYPE Clinic at Orygen Youth Health [24]. Orygen Youth Health is the state-funded specialist mental health service for 15- to 25-year-olds living in western metropolitan Melbourne, Australia. YMHS + BEF is being conducted at *headspace* in western metropolitan Melbourne [31], which is part of a national network of federally funded youth (12-to 25-year-olds) mental health clinics that is managed independently of Orygen Youth Health. It has a similar multidisciplinary outpatient staffing profile and services a similar catchment area to Orygen Youth Health. Therefore, the clientele of the two services are socio-demographically similar. Should the need arise, headspace patients have access to Orygen Youth Health’s after-hours crisis and inpatient services.

### Participants

All participants provide written informed consent. Participants comprise help-seeking young people who either self-refer or are referred to the Orygen Youth Health or headspace intake systems. Broad inclusion criteria are used to maximise the external validity of the trial:

- Aged 15–25 years, inclusive
- Ability to give informed consent and to comply with study procedures
- Fluency in English
- A Structured Clinical Interview for DSM-IV Axis II Disorders (SCID-II [32]) diagnosis of borderline personality disorder

Exclusion criteria are:

- Structured Clinical Interview for DSM-IV Axis I Disorders (SCID-I/P [33]) psychotic disorder within the past 12 months
- Lifetime SCID-I/P diagnosis of DSM-IV schizophrenia spectrum disorder, bipolar I or II disorder.
- Psychiatric condition due to a medical condition
- Severe disturbance, such that the person is unable to comply with either the requirements of informed consent or the study protocol
- Prior evidence-based treatment for borderline personality disorder
- Not meeting the clinical services’ eligibility criteria (for example, catchment area)

### Enrolment and randomisation

Patients at Orygen Youth Health are routinely screened using the 15 self-report borderline personality disorder items from the SCID-II Personality Questionnaire (SCID-II PQ) [32]. Patients at headspace are optionally screened using the SCID-II PQ. Published data from a previous study at Orygen Youth Health [34] indicate that a cut score of >12 has adequate sensitivity and good specificity and negative predictive value for BPD in youth. All patients scoring >12 are considered for eligibility, along with other trial inclusion criteria. In addition, patients who might screen negative for BPD (SCID-II PQ score ≤12) or who eluded screening but are identified by clinicians as meeting criteria for BPD using the SCID-II borderline personality disorder module [32] are also considered for eligibility, along with other trial inclusion criteria.

Participants are invited to participate by the trial coordinator, who contacts the young person (and their parent or legal guardian for those under 18 years) to introduce the study and to offer an appointment to obtain written consent. After the trial coordinator obtains written consent, a research assistant or the trial coordinator conducts the baseline assessment. All participants receive assertive case management, crisis care and general psychiatric care during the pre-screening and assessment phase. Once eligibility for the trial is confirmed, the trial coordinator randomly and consecutively assigns participants to one of the three interventions in a ratio of 1:1:1 using a password-protected computer program with a randomisation sequence that was computer-generated by an independent statistician. Treatment allocation uses randomised permuted blocking, and participants are stratified by age (cut point of age 18 years), sex and the Centre for Epidemiological Studies Depression Scale Revised (CESD-R) [35] score (cut point = 37, which is the mean score for borderline personality disorder patients on entry to HYPE), thus minimising these potentially confounding factors. Following randomisation to a treatment group, the trial coordinator then randomly assigns participants to the next available trial clinician using a separate randomisation sequence that was also computer-generated by an independent statistician. The trial coordinator contacts the participant and the treating service to inform them of the treatment group and clinician allocation. The clinician then contacts the participant to arrange the first appointment. Thus, the trial coordinator conducts the consent and randomisation procedures separately from the blinded research assistants, using independently derived, password-protected, computer-generated randomisation sequences.

The research assistants conduct all follow-up assessments. The study design and the treatment group assignment are concealed from the research assistants conducting the assessments. Blinding of the research assistants is achieved and maintained by having a specially written version of the trial protocol that removes all references to the three-group design and to any form of treatment. Most research assessments are conducted in the community or at an Orygen Youth Health assessment clinic where no community treatment takes place. Research assistant contact with clinical staff is minimised, and all trial participants, trial clinicians and other staff are regularly reminded of the importance of maintaining the blinding of the research assistants. The research assistants are aware that they are blind to elements of the study. Blinding of the research assistants will be tested at the end of their employment or the end of the study using an online self-report instrument designed specifically for the study. The study’s biostatistician is also blinded with respect to the randomisation.

### Interventions

The HYPE + CAT group receives up to 16 × 50 minute CAT sessions. Both the HYPE + BEF and YMH + BEF groups receive up to 16 × 50 minute sessions of befriending. Clinicians aim to deliver the 16 CAT or befriending sessions weekly, but the disorganised behaviour evident in many in this patient population means that it usually takes approximately 26 weeks to deliver them. Completion of 16 sessions marks the completion of all trial treatment. At the end of treatment or at any stage thereafter, participants can be referred for or seek additional treatment.

All participants receive case management and general psychiatric care as needed, and crisis and inpatient services accessed from the state mental health system, where indicated.

#### HYPE

The HYPE service model uses CAT as its theoretical model and common language. However, HYPE can be used with or without CAT as an individual psychotherapy. Key HYPE components include: (i) an explicitly collaborative approach with patients; (ii) assertive, ‘psychologically informed’ case management; (iii) flexible timing and location of sessions, including capacity for ‘outreach’ care in the community; (iv) active engagement and inclusion of families and carers; (v) use of a consistent, common and ‘plain language’ model across all aspects of care; (vi) psychoeducation for patients, families, carers, schools and others involved with the young person; (vii) integration of general psychiatric care within the same team, with specific assessment and treatment of co-occurring psychiatric syndromes (‘comorbidity’); (viii) access to after hours crisis and brief, goal-directed inpatient care; (ix) a focus on psychosocial recovery; (x) individual and group supervision of staff; (xi) maintenance of treatment fidelity. The rationale for this model has been formalised and published [24].

#### YMHS

Headspace provides high-quality, general mental health care delivered by specialist youth mental health clinicians within a multidisciplinary group practice setting. Clinicians diagnose mental disorders, develop a management plan, offer psychoeducation, conduct case management and offer general psychiatric care. General practitioner and/or psychiatric referrals and pharmacotherapy are initiated as indicated. Crisis and inpatient services are accessed from the state-based mental health system, as needed.

#### CAT

CAT is a time-limited, integrative psychotherapy that arose from a theoretical and practical integration of elements of psychoanalytic object relations theory and cognitive psychology, subsequently developing into an integrated model of development and psychopathology [23, 36]. CAT is practical and collaborative in style, with a particular focus upon understanding the individual’s problematic self-management and interpersonal relationship patterns and the thoughts, feelings and behavioural responses that result from these patterns. A central feature in CAT is the joint (patient–therapist) creation of a shared understanding of the patient’s difficulties and their developmental origins, using plain-language written and diagrammatic ‘reformulations’. These form the basis for understanding self-management and relationship problems both within and outside therapy, assist the patient in recognising and revising their dysfunctional relationship patterns and assist the therapist in avoiding or recovering from collusion with such relationship patterns.

CAT has particular theoretical advantages for early intervention for borderline personality disorder, especially because its integrative and ‘transdiagnostic’ approach encompasses the myriad co-occurring problems, which are the norm in this patient group, within the overall treatment model.

#### Befriending

Befriending is a manualised treatment that has been used in a wide range of psychotherapy trials [37] in order to control for many of the ‘common factors’ of psychotherapy, such as time in therapy, participant expectations and positive experiences of therapy. Befriending sessions are up to one hour in duration and are conducted weekly by a provisionally registered psychologist, in the same manner as a therapist would see a patient weekly. Each befriending session has the same format and consists of talking about neutral topics that interest the participant, such as vocational and educational activities, music, sport, books, cooking and pets. If the participant finds verbal interaction difficult, activities such as board games, walking or playing sports can be used with a view to using the activity as a tool to engage the participant in further neutral conversation during and after the activity. The therapist’s primary goals are to keep the participant engaged for the full duration of therapy and to keep the conversation or activity as close to a neutral ‘pleasant chat’ as possible. When emotionally loaded topics arise, such as symptoms or interpersonal problems, the therapist gently redirects the participant to more neutral topics.

Befrienders are trained and supervised by one of the investigators (JG) and another clinical psychologist, both of whom are expert in the intervention.

### Treatment integrity

All treatment sessions are audio-recorded to enable treatment integrity checks. CAT and befriending are both manualised treatments. Integrity (adherence, competency and separation) will be managed via regular expert supervision for CAT and befriending. Each CAT therapist will have two randomly selected CAT session recordings rated for adherence and competency, using a scale designed specifically for CAT [38]. A random selection of 50 % of befriending clinicians will have two randomly selected sessions rated for adherence and competency, using a scale designed specifically for befriending [37], and for treatment separation using the CAT scale. The befriending scale measures whether the therapist: (i) redirects from unresolved conflicts to a neutral topic, (ii) redirects from discussion about symptoms to a neutral topic, (iii) chooses the most neutral line of questioning, (iv) reacts minimally to loaded speech (for example, symptoms, conflicts), (v) redirects from identity issues to a neutral topic and (vi) predominantly engages the client in neutral conversations on day-to-day subjects.

The HYPE service model is manualised, but YMHS is not. Integrity of HYPE and YMHS will be maintained through the structure of the services and clinician supervision. HYPE also uses checklists of the tasks of care. Measures have been implemented to reduce ‘contamination’ of treatments and thereby to ensure treatment separation. HYPE and YMHS are governed and administered by separate organisations. YMHS clinicians have never worked in HYPE and do not have regular clinical contact with HYPE staff. Clinicians conducting the befriending have no training in CAT and are excluded from conducting further befriending if they commence CAT training at any time during the trial. CAT practitioners are permitted to have exposure to other clinical intervention techniques.

A random selection of 50 % of case managers will have two randomly selected sessions rated for treatment separation (to ensure that CAT is not being done covertly), using the above-mentioned CAT scale [38].

### Outcome measures

The primary endpoint of the trial is 12 months after baseline assessment. Assessments occur at 3, 6, 12 and 18 months after baseline. Table 1 lists the primary and secondary outcomes, along with subsidiary measures. Multiple methods are being employed to assess outcomes. These include independent observer rating, self-report, and Mobiletype© [39], a novel method of *in vivo* ecological momentary assessment.

**Table 1:** Schedule of outcome measures

	Time point (months)				
Measure	Baseline	3	6	12	18
Primary outcomes					
Inventory of Interpersonal Problems Circumplex Version (IIP-C)	✓	✓	✓	✓	✓
Social Adjustment Scale Self-Report (SAS-SR)	✓	✓	✓	✓	✓
Secondary outcomes					
Client Satisfaction Questionnaire (CSQ-8)	✓	✓	✓	✓	✓
Borderline Personality Disorder Severity Index (BPDSI-IV)	✓	✓	✓	✓	✓
Ecological momentary assessment of suicidal ideation and deliberate self-harm^a^	✓	✓	✓	✓	✓
Ecological momentary assessment of Positive and Negative Affect Scale (PANAS)^a^	✓	✓	✓	✓	✓
Beck Suicidal Ideation Scale (BSS)	✓	✓	✓	✓	✓
Suicide Attempt and Self-Injury Interview (SASII)	✓	✓	✓	✓	✓
Centre for Epidemiological Studies Depression Scale Revised (CESD-R)	✓	✓	✓	✓	✓
Montgomery–Aspberg Depression Rating Scale (MADRS/SIGMA)	✓	✓	✓	✓	✓
Alcohol Use Disorders Identification Test (AUDIT)	✓	✓	✓	✓	✓
Opiate Treatment Index (OTI - Section II)	✓	✓	✓	✓	✓
Subsidiary measures					
Difficulties in Emotional Regulation Scale (DERS)	✓	✓	✓	✓	✓
Quality of Life (AQoL-8D)	✓	✓	✓	✓	✓
Social and occupational functioning (SOFAS)	✓	✓	✓	✓	✓
Working Alliance Inventory (WAI)^b^	^b^	^b^	^b^		
Demographics	✓	✓	✓	✓	✓
Treatment information	✓	✓	✓	✓	✓
Diagnosis DSM-IV SCID-I/P	✓	✓	✓	✓	✓
Diagnosis DSM-IV SCID-II	✓			✓	✓

^a^Ecological momentary assessment uses the Mobiletype© mobile phone system to capture suicidal ideation, deliberate self-harm and affective state *in vivo*. Assessments occur six times per day for six days

^b^Administered at the commencement of treatment, mid-treatment and post final treatment session

The primary outcome is adaptive functioning, defined by measures of interpersonal problems [40] and social adjustment [41]. This was chosen in light of the evidence that impairments in adaptive functioning among individuals with borderline personality disorder tend to be persistent and refractory to current treatments [14]. The Inventory of Interpersonal Problems Circumplex Scales (IIP-C) [40] has 64 self-report items, rated using a five-point scale ranging from 0 (not at all) to 4 (extremely). It contains eight scales (Domineering, Vindictive, Cold, Socially Inhibited, Overly Accommodating, Non-assertive, Self-Sacrificing, Intrusive). The Social Adjustment Scale Self-Report (SAS-SR) [41] has 54 self-report items, scored on a five-point scale, measuring instrumental and expressive role performance over the previous 2 weeks. It yields scores in six domains (Primary Relationship, Work, Social and Leisure, Extended Family, Parental (own children) and Family Unit (partner or children)) and a total score, calculated by averaging all applicable items. The SAS-SR contains ‘skip-outs’, so that non-applicable items are omitted, making it appropriate for the age range of participants in the proposed study.

Secondary outcomes measure client satisfaction [42], borderline personality disorder features [43], suicidal ideation and deliberate self-harm [39, 44, 45], affective instability [39, 46], depression [35, 47] and substance use [48, 49]. Subsidiary outcome measures include emotion regulation [50], quality of life [51], a summary measure of social and occupational functioning [52], therapeutic alliance [53] and demographic, treatment and diagnostic information [32, 54].

### Safety

Clinical governance for trial participants rests with the service to which they are allocated (Orygen Youth Health or *headspace*). Treatment continues for approximately 26 weeks, unless the participant meets the trial’s withdrawal criteria. Withdrawal from the trial’s treatment program will occur if:

- Participation in the study interferes with the appropriate clinical management of risk of self-harm or harm to others. This decision is initiated by the treating clinical team, in consultation with the principal investigator.
- The participant meets the operational criteria for Orygen Youth Health’s first-episode psychosis program, or DSM-IV operational criteria for bipolar I or II disorder. These will be assessed by an independent psychiatrist (or trainee psychiatrist).
- Consent is withdrawn

### Data integrity

The research assistants who conduct follow-up assessments are psychology graduates who are trained and supervised by the chief investigator/consultant psychiatrist and the trial coordinator/clinical psychologist. Assessment interviews are audio recorded, and ten percent of assessments at each time point are randomly selected for inter-rater reliability.

Data entry verification is undertaken on a randomly selected twenty percent of cases at each time point, with an *a priori* acceptable rate of 0.5 %. The statistician who will perform the analyses is also blind to treatment group allocation.

### Statistical analyses and sample size determination

To determine whether there are group differences on the primary and secondary outcome measures at 12 months, a series of mixed effects model repeated measures analysis of variances models (MMRM) will be employed. The within groups factor will be time, and the group will serve as the between subjects factor. MMRM is a preferred method for analysing clinical trial data in psychiatry in comparison with the more traditional repeated measures analysis of variance and analysis of covariance models [55]. MMRM is advantageous because it includes all existing data in the model, there is no imputation or substitution of missing data, dispersion and correlation can occur at all time points, and sensitivity analysis can be successfully conducted. Within these models, planned comparisons will be used to determine whether HYPE + CAT is superior to the other two interventions, and whether the HYPE service model leads to better outcomes compared with YMHS.

Power and sample size calculations have been based on a one-way analysis of covariance model with baseline values as the covariate, and the outcome measures as the dependent variables. It is assumed that the baseline covariate will account for 50 % of the variance in an outcome measure. Based on our published data [29, 56], we expect to find medium treatment effects on the primary outcome measures of interpersonal problems and social adjustment. Alpha has been set at 0.05 and power (1-β) for the study at 0.80. Given these parameters, we require 40 participants per group (120 in total) with at least two data points. Based upon the 14 % attrition rate in our previous randomised control trial we intend to recruit at least 45 cases per group.

## Discussion

The current trial extends previous research and addresses the following questions. What is the comparative effectiveness of three different levels of complexity of early intervention for borderline personality disorder? What is the comparative effectiveness of a specialised early intervention service model over and above general youth mental health care in early intervention for borderline personality disorder? What is the contribution of individual cognitive analytic therapy over and above specialised general clinical care in early intervention for borderline personality disorder? Is early intervention for borderline personality disorder effective for emerging adults as well as for adolescents?

In order to answer the above questions, the trial separately evaluates early intervention, independent of indicated prevention, by only including full-syndrome borderline personality disorder. This has an added advantage of reducing heterogeneity among the sample. The trial also extends the age range of new onset cases studied from 15–18 to 15–25 years. It also focuses upon functional outcomes, reflecting recent findings that this aspect of borderline personality disorder is stably poor over long-term follow-up, refractory to current treatments, and should be the primary focus of treatment outcome studies for borderline personality disorder [6, 12, 14].

While the trial design has attempted to address many problems identified in previous studies, some problems are anticipated. Safety measures for the trial mean that participants who drop out of the trial intervention but who require continuing treatment in a mental health service will be eligible for referral to Orygen Youth Health, meaning that they might receive HYPE + CAT, which is the default treatment offered at Orygen. This will be examined in secondary analyses.

The results of this study will be highly informative. Should the more complex HYPE + CAT treatment prove to be most effective, it will further support the development of specialised early intervention services for borderline personality disorder in the mental health system. However, should individual cognitive analytic therapy be found to confer little or no benefit in terms of outcome over and above the specialised HYPE service model, this would have major implications for translation into clinical services. Service organisation around the HYPE model requires knowledge of the principles of CAT but does not require the intensive 2-year training course to become a CAT practitioner. Should YMHS be found to be as effective as the specialised treatment, this would have significant implications for scaling up of services for early intervention for borderline personality disorder. Such broad-based, youth specialist services have the greatest potential to be implemented across mental health systems in a potentially highly cost-effective manner. Such a finding would also signal to the general community of youth mental health clinicians that borderline personality disorder can and should be a key target for mainstream services.

## Trial status

Trial recruitment commenced in March 2011 and is continuing. One hundred and thirty-five participants have been enrolled. Publication of this manuscript has been held until as late as possible in the trial in order to minimise the risk of un-blinding the research assessors.

## Abbreviations

BEF: befriending
CAT: cognitive analytic therapy
CESD-R: Centre for Epidemiological Studies Depression Scale Revised
DBT-A: Dialectical Behaviour Therapy for Adolescents
DSM-5: Diagnostic and Statistical Manual of Mental Disorders, fifth edition
ERT: Emotion Regulation Training
EUC: enhanced usual care
GCC: good clinical care
GCP: Good Clinical Practice
HYPE: Helping Young People Early
ICD-11: International Classification of Diseases, 11th revision
MBT-A: Mentalisation-Based Treatment for Adolescents
MMRM: mixed effects model repeated measures analysis of variances
RCT: randomised controlled trial
SCID-II: Structured Clinical Interview for DSM-IV Axis II Disorders
SCID-II PQ: SCID-II Personality Questionnaire
SCID-I/P: Structured Clinical Interview for DSM-IV Axis I Disorders
TAU: treatment as usual
YMHS: youth mental health service
